# Glycine and N-Acetylcysteine (GlyNAC) Combined with Body Weight Support Treadmill Training Improved Spinal Cord and Skeletal Muscle Structure and Function in Rats with Spinal Cord Injury

**DOI:** 10.3390/nu15214578

**Published:** 2023-10-28

**Authors:** Xin Xu, Hua-Yong Du, Zuliyaer Talifu, Chun-Jia Zhang, Ze-Hui Li, Wu-Bo Liu, Yi-Xiong Liang, Xu-Luan Xu, Jin-Ming Zhang, De-Gang Yang, Feng Gao, Liang-Jie Du, Yan Yu, Ying-Li Jing, Jian-Jun Li

**Affiliations:** 1 School of Rehabilitation, Capital Medical University, Beijing 100069, China; doyourself0506@163.com (X.X.);; 2 School of Population Medicine and Public Health, Chinese Academy of Medical Sciences/Peking Union Medical College, Beijing 100730, China; 3 Department of Orthopedics, Qilu Hospital of Shandong University, Jinan 250100, China

**Keywords:** spinal cord injury, GlyNAC, BWSTT, skeletal muscle atrophy, nerve function, skeletal muscle fibers

## Abstract

Skeletal muscle atrophy is a frequent complication after spinal cord injury (SCI) and can influence the recovery of motor function and metabolism in affected patients. Delaying skeletal muscle atrophy can promote functional recovery in SCI rats. In the present study, we investigated whether a combination of body weight support treadmill training (BWSTT) and glycine and N-acetylcysteine (GlyNAC) could exert neuroprotective effects, promote motor function recovery, and delay skeletal muscle atrophy in rats with SCI, and we assessed the therapeutic effects of the double intervention from both a structural and functional viewpoint. We found that, after SCI, rats given GlyNAC alone showed an improvement in Basso–Beattie–Bresnahan (BBB) scores, gait symmetry, and results in the open field test, indicative of improved motor function, while GlyNAC combined with BWSTT was more effective than either treatment alone at ameliorating voluntary motor function in injured rats. Meanwhile, the results of the skeletal muscle myofiber cross-sectional area (CSA), hindlimb grip strength, and acetylcholinesterase (AChE) immunostaining analysis demonstrated that GlyNAC improved the structure and function of the skeletal muscle in rats with SCI and delayed the atrophication of skeletal muscle.

## 1. Introduction

Spinal cord injury (SCI) is a serious and disabling condition that can severely impair the motor, sensory, and autonomic functions of affected patients [1,2]. SCI results in the loss of central nervous system innervation of skeletal muscles; in particular, a complete SCI results in the permanent paralysis of skeletal muscles below the injured segment, leading to skeletal muscle atrophy [3,4]. Skeletal muscle accounts for approximately 40% of body weight and, in addition to controlling, protecting, and supporting body movement, it also plays an essential role in metabolism [5,6]. Skeletal muscle is a highly metabolically active tissue and consumes approximately 70% of the body’s glucose [7]. Skeletal muscle atrophy after SCI leads to both localized and systemic metabolic dysfunction [8]. Following SCI, skeletal muscle undergoes a series of pathophysiological changes. For instance, the proportion of adipose tissue in skeletal muscle is increased [9], while skeletal muscle cell apoptosis is also common after SCI, resulting in reduced skeletal muscle strength and endurance [10,11,12]. Muscle mass also decreases rapidly, ranging from 20% to 55% after complete SCI and 20% to 30% after incomplete SCI [6]. The average muscle cross-sectional area (CSA) at 6 weeks is reduced by 18% to 46% after SCI [11]. Concomitantly, there is a significant conversion from slow type I to fast type II fibers in skeletal muscle after SCI [5,13]. This is especially true for type IIx muscle fibers, which leads to a decrease in the ability of patients to fight fatigue [14]. These observations underscore the importance of early treatment after SCI for delaying or preventing skeletal muscle atrophy.

Skeletal muscle atrophy due to the loss of central nervous system innervation after SCI results from a lack of nutrient sources and excessive protein breakdown in skeletal muscle. Protein catabolism is greater than anabolism in skeletal muscle atrophy [10], and amino acid supplementation to promote skeletal muscle protein synthesis is an effective means for preventing skeletal muscle atrophy and weakness [15]. Glutathione (GSH) plays a significant role in defending against oxidative stress injury in the spinal cord and skeletal muscle after SCI; however, its levels are decreased after injury [16]. GlyNAC, a combination of glycine and N-acetylcysteine, can reportedly improve the body’s antioxidant capacity by moderately promoting the autonomous synthesis of GSH [17]. Through improving GSH deficiency, GlyNAC can reduce oxidative stress levels and mitigate mitochondrial dysfunction during the aging process [18,19]. The above findings indicate that the application of GlyNAC may represent a potential therapeutic approach for counteracting the oxidative stress injury secondary to SCI, slowing down skeletal muscle atrophy and dysfunction due to SCI, and promoting functional recovery. However, whether GlyNAC can exert such ameliorative effects in skeletal muscle atrophy after SCI has yet to be investigated. 

Body weight support treadmill training (BWSTT) is a clinical treatment commonly used to enhance the motor ability of patients with SCI and can, to a certain extent, alleviate SCI-induced muscle atrophy in these patients [20,21]. BWSTT can improve skeletal muscle sensitivity and reduce insulin resistance after SCI [22]. Accordingly, in this study, we investigated whether combined intervention with GlyNAC and BWSTT could promote neurological recovery, thereby delaying skeletal muscle atrophy and improving neurological function following injury to the spinal cord.

## 2. Materials and Methods

### 2.1. Animals and Grouping

**Figure 1:**
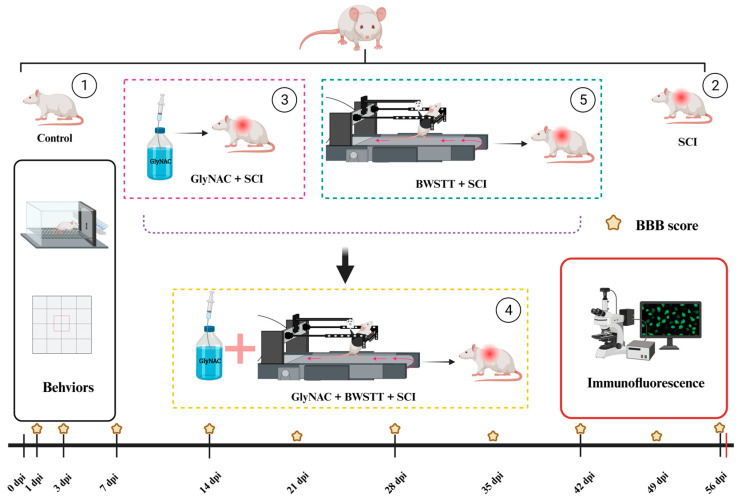
Grouping, experimental procedure, and time points. The rats were randomly divided into 5 groups, in which the SCI group, GlyNAC + SCI group, GlyNAC + BWSTT + SCI group, and BWSTT + SCI group underwent modeling surgery for spinal cord injury at the level of T10. GlyNAC was administered by gavage 1 day after modeling and the BBB score evaluation was performed on days 1 and 3 after modeling to determine whether the modeling was successful. Body weight was measured once before modeling and then once weekly for 8 consecutive weeks, BBB score evaluation was performed weekly for 8 consecutive weeks after modeling, and other relevant behavioral assessments were performed at 2, 4, 6, and 8 weeks post-injury; for immunofluorescence assays, samples were collected 8 weeks after modeling. BBB: Basso–Beattie–Bresnahan; GlyNAC: glycine and N-acetylcysteine; SCI: spinal cord injury; BWSTT: body weight support treadmill training.

Fifty female Sprague Dawley (SD) rats (SPF grade, 10 weeks of age) were used in this study and were purchased from Beijing Viton Lihua Laboratory Animal Technology Co., Ltd., Beijing, China. Animals that showed any movement of the hindlimbs one day and three days after the operation were excluded from the study. The rats that met the criteria were divided into the following five groups: a control group, an SCI group, a GlyNAC + SCI group, a BWSTT + SCI group, and a GlyNAC + BWSTT + SCI group. The grouping strategy and experimental protocol are shown in Figure 1.

### 2.2. Generation of the SCI Model and GlyNAC/BWSTT Intervention

Before the start of the modeling procedure, the rats in each group underwent two weeks of acclimatization in the animal house. After weighing, the rats were anesthetized with isoflurane and were placed in the prone position. After shaving and sterilizing, a 6 cm incision was made longitudinally with T10 as the center to fully expose the vertebral plates at T9–T11, and the T10 vertebral plate was excised to expose the spinal cord of the T10 segment. Subsequently, the T9 and T11 vertebral plates were clamped together. The spinal cord at the site to be struck was rinsed with physiological saline and the rats were positioned on a spinal cord percussion device (Precision Systems and Instrumentation IH Spinal Cord Percussion Device, Mexico, Fairfax Station, VA, USA). The percussion hammer was positioned at a height of 10–12 cm and the percussion force was set to 250 kdynes, resulting in a moderate SCI model. Swinging of the tail served as the initial confirmation of the success of the modeling. The surgical incision was closed and 1.5 mL of saline and 1 mL of penicillin solution were injected subcutaneously for rehydration and antibacterial treatment. After the operation, the rats were kept warm until they awoke, at which point they were returned to the corresponding rearing cages.

GlyNAC intervention was started at 10:00 am on the first postoperative day. A GlyNAC solution (200 mg/kg, an experimentally tested dose) was administered by gavage using a gavage needle (Heng’ao Biologicals, Shanghai, China). The gavage intervention was carried out for a total of 4 weeks. Body weight was evaluated once a week and the dosage was adjusted according to the change in body weight. 

BWSTT was started on day 14 after modeling and the rats were allowed to adapt to the BWSTT equipment before training. The daily training time was 15 min and the speed of the BWSTT equipment was set to 5 cm/s. The model rats were initially helped by the trainer to allow them to adapt to the two-legged walking mode on the belt so that the hindlimbs could be fully exercised. Bladder care was performed twice a day during the first week after the injury. If a rat had hematuria, penicillin injections were continued and the rat was closely monitored. During the second week after injury, bladder care was performed once daily.

### 2.3. Basso–Beattie–Bresnahan (BBB) Locomotor Rating

The BBB scores of the rats were evaluated on days 1 and 3 after surgery. Rats that showed any movement of the hip or the knee and ankle joints in the lower limbs were excluded from the experiment. Two observers blinded to the experimental grouping were selected to perform the BBB score evaluation and complete the recordings after modeling. The evaluation required the rats to move freely in an open field for 15 min, and the two observers performed the scoring and recorded the scores once a week for 8 weeks. The BBB scores ranged from 0 to 21.

### 2.4. Gait Analysis

The motor function of the rats, including gait data during walking, was assessed using a digital footprint analysis system (DigiGaitTM, Inc., Mouse Speciics, Inc., Framingham, MA, USA). During the evaluation, the running table was operated at a speed of either 5 or 10 cm/s, and at least five consecutive gait cycles were captured with a high-speed video camera (Basler A602 camera, 150 fps, Basler AG, Ahrensburg, Germany). For each rat, three images were recorded at a time. The gait parameters in the obtained images were digitally analyzed with DigiGait analysis software (Digigait 12.4) and the average value for each evaluation index was separately recorded. The main indexes selected for analysis were as follows: (1) gait symmetry: (right forelimb stepping frequency + left forelimb stepping frequency)/(right hindlimb stepping frequency + left hindlimb stepping frequency); (2) coefficient of variation of hind paw area (CV: 100× standard deviation/mean); (3) forelimb weight support: (width of each front paw + average touching width of front paw)/average width of the animal, and the width was calculated as the widest part of the body from the tip of the nose to the tail as the center axis; and (4) hind stance width: width between the two hindlimbs.

### 2.5. Open Field Test

The voluntary locomotor function of the rats was evaluated using a Cleversys TopScanLite device (Cleversys, VA, USA). The rats were placed in the evaluation environment 30 min before assessment. During the evaluation, the rats were placed in an open field (40 × 40 cm), and the trajectory of the rats in the open field, as well as some related parameters, was recorded for 10 min using a high-speed camera. The number of times the rats crossed the central area, the total distance traveled, and the average velocity of each rat during the experiment were recorded to assess the autonomic function and the emotional state of the animals. Between rats, the field area was wiped clean and disinfected with alcohol to prevent the odor of the previous rat from affecting the results.

### 2.6. Hindlimb Grip

The neuromuscular function of the hindlimbs of rats was evaluated at 4 and 8 weeks post-SCI using a grip strength meter (Columbus Instruments, Columbus, OH, USA), which provides an objective assessment of the grip strength of the rats during walking. Before the evaluation, the rats were allowed to adapt to grasping the specially designed tensile bar device with both feet. During the test, the rats were allowed to grasp the tensile bar bipedally and were then slowly pulled away from the bar until the paws were released; the peak force generated at this time was recorded. The three stable recorded values were averaged for comparisons among the five groups.

### 2.7. Electrophysiologic Detection of Motor Neuron Excitability

An eight-channel electrophysiologic recorder, PowerLab (Electronic Stimulator, ADInstruments, New South Wales, Australia), was used to evaluate the H-reflex in the rats. The animals were fixed on an operating table, anesthetized with 2% isoflurane, and the tibial nerve was stimulated by inserting an electrode needle into the medial side of the ankle joint, which induced plantarflexion. The electrogram was obtained after signal amplification and filtering. Twenty consecutive electrical stimulations were applied at the frequencies of 0.1, 0.5, 1, 2, and 5 Hz, respectively. The PowerLab software LabChart (Version 8.0, ADInstruments, Dunedin, New South Wales, Australia) was used for analysis and the last 15 of the 20 filters were selected for H-wave analysis. The rate-dependent depression (RDD) of the H-reflex was obtained by dividing the mean value of the peak H-wave at 0.1 Hz by the mean value of the peak H-wave at 0.5, 1, 2, and 5 Hz, respectively. The results were compared among the control, SCI, and GlyNAC + SCI groups.

### 2.8. Immunofluorescence Staining of the Spinal Cord and Skeletal Muscle

Eight-week-old rats were anesthetized with 2% pentobarbital sodium, fixed on the operating table, and the thoracic cavity of the rats was opened to fully expose the heart. A perfusion needle was inserted into the left ventricle and the rats were first perfused with 0.9% saline through the aorta and then with 4% paraformaldehyde solution for fixation. The lumbar enlargement (L2~L6) was subsequently fixed in tissue fixative (G1101, ServiceBio, Wuhan, China) while the gastrocnemius, soleus, and tibialis anterior tissues were fixed in muscle fixative (G1111, ServiceBio, Wuhan, China). After 24 h of fixation, spinal cord and skeletal muscle tissues were dehydrated using a graded ethanol series and paraffin-embedded. The tissues to be stained were circled with a histochemical pen, and the sections were sealed in 3% hydrogen peroxide at room temperature for 25 min protected from light, washed three times with PBS (pH 7.4), blocked with a drop of 3% BSA, and incubated with primary antibodies (Table 1) after the removal of the blocking solution. After rewarming and incubation with the corresponding HRP-labeled secondary antibody, the sections were counterstained with DAPI, washed with PBS, and sealed with an autofluorescence quenching agent (G1221, ServiceBio, Wuhan, China). The samples were analyzed and imaged under a fluorescence microscope (Eclipse C1, Nikon, Tokyo, Japan) and the number of neurons and motor neurons in the spinal cord, the percentage of slow skeletal muscle fibers, and the fluorescence intensity and fluorescence area of AChE were determined.

### 2.9. Hematoxylin and Eosin (H&E) Staining

The gastrocnemius, soleus, and tibialis anterior tissues previously stored in muscle fixative (G1111, ServiceBio, Wuhan, China) were paraffin-embedded, sliced into 5 μM thick sections, and stained with hematoxylin–eosin reagent (Servicebio). The stained sections were viewed through a light microscope and the myofiber density and myofiber CSA size of the muscle bundles in the different skeletal muscles were determined using ImageJ (ImageJ 1.46r).

### 2.10. Statistical Analysis

Statistical analysis was performed using SPSS (version 19.0, IBM, Armonk, NY, USA). Comparisons between two groups were made using two independent samples *t*-tests, while comparisons among three or more groups were made using one-way ANOVA with correction for *p*-values using the Bonferroni post hoc test. Data are shown as means ± standard deviation and *p*-values < 0.05 were considered significant. GraphPad Prism (version 9.3.0.463, LLC, San Diego, CA, USA) was used for graph plotting.

## 3. Results

### 3.1. GlyNAC Significantly Improved BBB Scores and Enhanced Gait Symmetry in Rats

**Figure 2:**
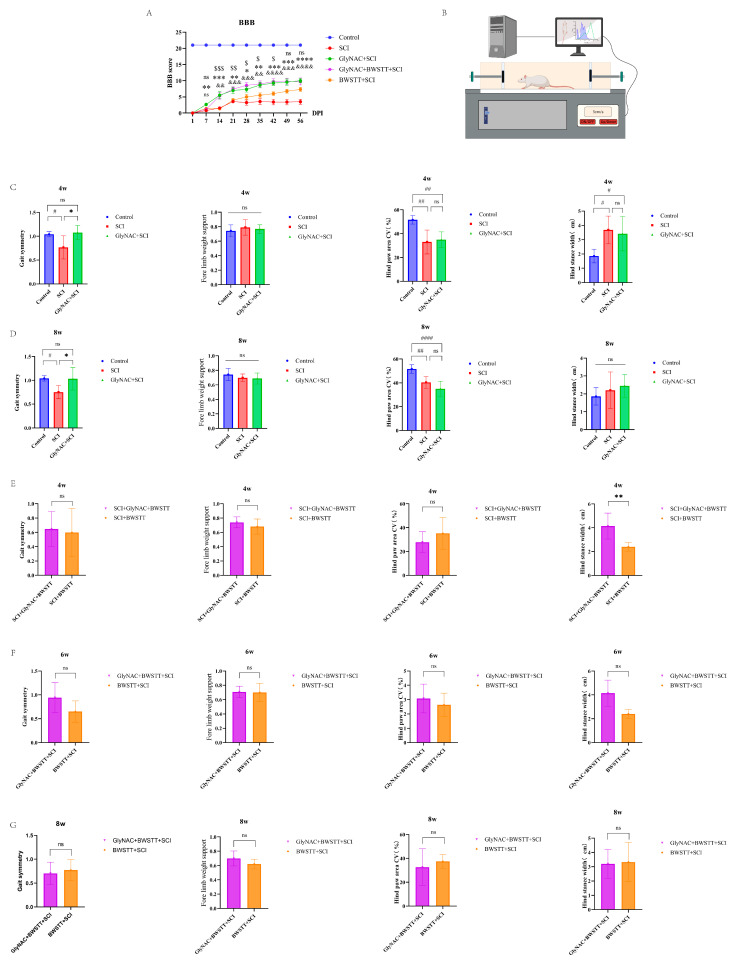
BBB scores and gait analysis. (**A**) BBB scores on day 1 and 1–8 weeks after modeling in the control, SCI, GlyNAC + SCI, GlyNAC + BWSTT + SCI, and BWSTT + SCI groups. $, *, and & indicate significant differences between the GlyNAC + BWSTT + SCI group and the SCI group, the GlyNAC + SCI group and the SCI group, and the GlyNAC + BWSTT + SCI group and the BWSTT + SCI group, respectively; ns denotes not significant (one-way ANOVA, Bonferroni post hoc test). $, * indicate *p* < 0.05. $$, **, && indicate *p* < 0.01. $$$, ***, &&& indicate *p* < 0.001. ****, &&&& indicate *p* < 0.0001. (**B**) Schematic diagram of gait data evaluation in rats, which was performed using a digital footprint analysis system to appraise motor function. (**C**,**D**) The results of gait data analysis—gait symmetry, forelimb weight support, coefficient of variation of hind paw area, and hind stance width—for the control, SCI, and GlyNAC + SCI groups at 2, 4, 6, and 8 weeks after modeling. * and # indicate significant differences between the SCI and GlyNAC + SCI groups, the control and SCI groups, and the control and GlyNAC + SCI groups, respectively; ns denotes not significant (one-way ANOVA, Bonferroni post hoc test). #, * indicates *p* < 0.05, ## indicates *p* < 0.01, #### indicates *p* < 0.0001. (**E**–**G**) The results of the gait data analysis—gait symmetry, forelimb weight support, coefficient of variation of hind paw area, and hind stance width—for the GlyNAC + BWSTT + SCI and BWSTT + SCI groups at 4, 6, and 8 weeks after modeling. * indicates a significant difference between the GlyNAC + BWSTT + SCI and BWSTT + SCI groups, ns indicates not significant (one-way ANOVA, Bonferroni post hoc test). ** indicates *p* < 0.01. BBB: Basso-Beattie-Bresnahan; GlyNAC: glycine and N-acetylcysteine; SCI: spinal cord injury; BWSTT: body weight support treadmill training.

Figure 2A shows the BBB score trend at 8 weeks post-injury among the five groups. By the end of the first week post-injury, a significant difference in BBB scores could already be observed between the GlyNAC + SCI and SCI groups, and this difference showed a gradual but increasing trend (*p* = 0.0057, *p* = 0.0007, *p* = 0.0022, *p* = 0.0113, *p* = 0.0027, *p* = 0.0002, *p* = 0.0003, and *p* = 0.0001 for weeks 1–8, respectively). From the beginning of week 2 to week 8, the GlyNAC + BWSTT + SCI group showed a significant improvement in BBB scores compared with the SCI group, with the difference again showing a progressively increasing trend (*p* = 0.0014, *p* = 0.0004, *p* = 0.001, *p* = 0.0015, *p* = 0.0001, *p* = 0.0003, and *p* = 0.0001 for weeks 2–8, respectively). From the beginning of week 2 to week 6, the GlyNAC + BWSTT + SCI group showed a significant improvement in BBB scores relative to the BWSTT + SCI group (*p* = 0.0003, *p* = 0.0051, *p* = 0.0286, *p* = 0.0458, and *p* = 0.0159 for weeks 2–6, respectively); however, no difference in BBB scores was detected between the GlyNAC + BWSTT + SCI and GlyNAC + SCI groups.

A schematic diagram of the gait analysis is shown in Figure 2B. We first compared the gait data for the control, SCI, and GlyNAC + SCI groups (Figure 2C,D) to assess the effect of GlyNAC on the gait of SCI model rats. The time points selected for this analysis were 4 and 8 weeks after injury and the evaluation speed was 5 cm/s. At the 4-week time point, the GlyNAC + SCI group (1.075 ± 0.2427) exhibited better gait symmetry than the SCI group (0.7661 ± 0.1496; *p* = 0.0212), and there was a significant decrease in gait symmetry in the SCI group compared with that in the control group (*p* = 0.0315). No significant difference in forelimb weight support was observed between the GlyNAC + SCI and SCI groups (*p* = 0.9025). In contrast, the coefficient of variation of hind paw area decreased significantly in both the SCI and GlyNAC + SCI groups compared with that in the control group (*p* = 0.0012 and *p* = 0.003, respectively); however, there was no significant difference in this parameter between the two groups (*p* = 0.8837). Compared with the control group, the hind stance width was significantly increased in both the SCI and GlyNAC + SCI groups (*p* = 0.0104 and *p* = 0.0281, respectively), but it did not differ significantly between the two groups (*p* = 0.8704). At 8 weeks post-injury, the GlyNAC + SCI group (1.0306 ± 0.2347) showed better gait symmetry than the SCI group (0.7517 ± 0.1395; *p* = 0.0261), while the SCI group exhibited a significant decrease in gait symmetry compared with that in the control group (*p* = 0.0114). There was no significant difference in forelimb weight support between the GlyNAC + SCI and SCI groups (*p* = 0.9714). The coefficient of variation of hind paw area was significantly decreased in both the SCI and GlyNAC + SCI groups relative to that seen in the control group (*p* = 0.0042 and *p* = 0.0001, respectively), but it was similar between the two groups (*p* = 0.1894). The hind stance width did not differ significantly between the SCI group and the control group (*p* = 0.38), between the GlyNAC + SCI group and the control group (*p* = 0.71), or between the SCI group and the GlyNAC + SCI group (*p* = 0.8361). Next, we compared the gait data of the GlyNAC + BWSTT + SCI and BWSTT + SCI groups when the running table speed was set at 10 cm/s (Figure 2E–G). The results showed that, after 4 weeks, there was a significant increase in hind stance width in the GlyNAC + BWSTT + SCI group compared with that in the BWSTT + SCI group (*p* = 0.0043); however, no difference in gait symmetry, forelimb weight support, or coefficient of variation of hind paw area was found between the two groups (*p* = 0.7843, *p* = 0.2963, and *p* = 0.2778, respectively); similarly, no difference in gait symmetry, forelimb weight support, the coefficient of variation of hind paw area, or hind stance width was detected between the two groups at 6 and 8 weeks post-injury.

Combined, these results suggested that GlyNAC can improve motor function and gait symmetry over time in rats after SCI as determined by the higher BBB scores in rats administered the GlyNAC/BWSTT combination treatment compared with those of animals receiving BWSTT alone; however, when the gait data were included, the therapeutic effect of the combination treatment was not significantly greater than that of the single treatment.

### 3.2. Open Field Test Results

**Figure 3:**
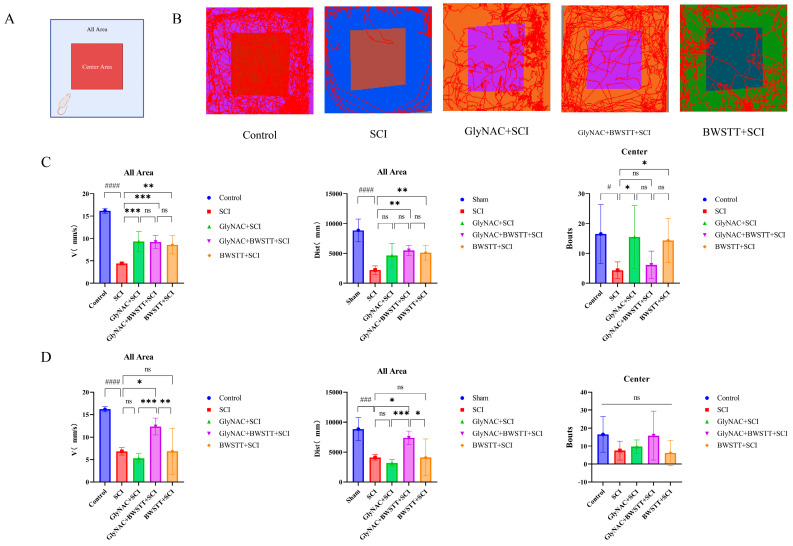
Results of the open field test. (**A**) Schematic representation of the whole field and the central area of the open field. (**B**) The trajectory maps of the control, SCI, GlyNAC + SCI, GlyNAC + BWSTT + SCI, and BWSTT + SCI groups in the open field. (**C**,**D**) Results of the statistical analysis of velocity, total distance moved, and the number of entries into the central area at 4 and 8 weeks after modeling for the control, SCI, GlyNAC + SCI, GlyNAC + BWSTT + SCI, and BWSTT + SCI groups. * indicates significant differences between the GlyNAC + SCI, GlyNAC + BWSTT + SCI, and BWSTT + SCI groups and the SCI group, # indicates significant difference between the control group and the SCI group, and ns indicates not significant (one-way ANOVA, Bonferroni post hoc test). *, # indicate *p* < 0.05. ** indicates *p* < 0.01. ***, ### indicate *p* < 0.001. #### indicate *p* < 0.0001., respectively. GlyNAC: glycine and N-acetylcysteine; SCI: spinal cord injury; BWSTT: body weight support treadmill training.

The results of the comparisons among the five groups of rats in the open field test relating to distance moved, velocity, and the number of times crossing the central area are shown in Figure 3. We found that at 4 weeks post-surgery, rats in the GlyNAC + BWSTT + SCI and BWSTT + SCI groups had moved significantly longer distances in the open field compared with the SCI group (*p* = 0.0036 and *p* = 0.0067, respectively). However, no significant difference in distance traveled was recorded between the GlyNAC + BWSTT + SCI group and the GlyNAC + SCI group (*p* = 0.7892) or the BWSTT + SCI group (*p* = 0.999). Rats in the GlyNAC + SCI, GlyNAC + BWSTT + SCI, and BWSTT + SCI groups moved at significantly greater velocities compared with those in the SCI group in the open field (*p* = 0.0007, *p* = 0.0009, and *p* = 0.0014, respectively); there was no significant difference in velocity between the GlyNAC + BWSTT + SCI group and the GlyNAC + SCI group (*p* > 0.05) or between the GlyNAC + BWSTT + SCI group and the BWSTT + SCI group (*p* = 0.9997). Additionally, rats in the GlyNAC + SCI and BWSTT + SCI groups traveled through the central area significantly more frequently than those in the SCI group (*p* = 0.0173 and *p* = 0.0293, respectively); no significant difference in this parameter was observed between the GlyNAC + BWSTT + SCI group and the GlyNAC + SCI group (*p* = 0.1015) or between the GlyNAC + BWSTT + SCI group and the BWSTT + SCI group (*p* = 0.1573). At 8 weeks post-injury, animals in the GlyNAC + BWSTT + SCI group had traveled significantly longer distances in the open field compared with those in the SCI group (*p* = 0.0228); similarly, rats in the GlyNAC + BWSTT + SCI group moved significantly longer distances in comparison with those in the GlyNAC + SCI group (*p* = 0.0007) or the BWSTT + SCI group (*p* = 0.0106); meanwhile, the velocities were significantly greater in the GlyNAC + BWSTT + SCI group than in the SCI (*p* = 0.0242), GlyNAC + SCI (*p* = 0.0005), and the BWSTT + SCI groups (*p* = 0.0073); there was no significant difference in the numbers of times the rats traveled through the central area among the five groups at the 8-week time point.

### 3.3. GlyNAC Combined with BWSTT Significantly Improved Hindlimb Grip in the Rats

**Figure 4:**
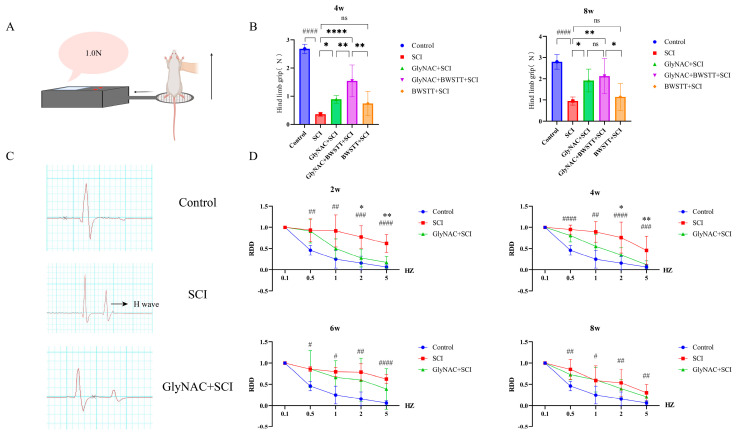
Hindlimb grip and electrophysiological analysis. (**A**) Schematic of the grip force detection method. (**B**) Results of the analysis of hindlimb grip measurement data at 4 and 8 weeks after modeling in the control, SCI, GlyNAC + SCI, GlyNAC + BWSTT + SCI, and BWSTT + SCI groups. (**C**) Skeletal muscle electrophysiological detection waveforms in the control, SCI, and GlyNAC + SCI groups. (**D**) Results of the analysis of skeletal muscle electrophysiology RDD data at 2, 4, 6, and 8 weeks after modeling in the control, SCI, and GlyNAC + SCI groups. * indicates significant differences between the GlyNAC + SCI group and the SCI group, # indicates significant differences between the control group and the SCI group, and ns indicates not significant (one-way ANOVA, Bonferroni post hoc test). * and # indicate *p* < 0.05. ** and ## indicate *p* < 0.01. ### indicates *p* < 0.001. **** and #### indicate *p* < 0.0001. GlyNAC: glycine and N-acetylcysteine; SCI: spinal cord injury; BWSTT: body weight support treadmill training; RDD: rate−dependent depression.

The differences in grip strength among the five groups are depicted in Figure 4A,B. The results showed that at 4 weeks post-SCI, the hindlimb grip was significantly stronger in the GlyNAC + SCI and GlyNAC + BWSTT + SCI groups than in the SCI group (*p* = 0.0219 and *p* < 0.0001, respectively), but it did not differ significantly between the BWSTT + SCI group and the SCI group (*p* = 0.1004). The GlyNAC + BWSTT + SCI group displayed a more significant improvement in hindlimb grip than either the GlyNAC + SCI group (*p* = 0.0095) or the BWSTT + SCI group (*p* = 0.0017); at 8 weeks, meanwhile, there was a more significant improvement in hindlimb grip in the GlyNAC + SCI and GlyNAC + BWSTT + SCI groups than in the SCI group (*p* = 0.0242 and *p* = 0.0028, respectively); however, there was no significant difference in hindlimb grip between the BWSTT + SCI and SCI groups (*p* = 0.8287) and the GlyNAC + BWSTT + SCI group had a more significant enhancement in hindlimb grip compared with the BWSTT + SCI group (*p* = 0.0347); there was no significant difference in hindlimb grip between the GlyNAC + BWSTT + SCI and GlyNAC + SCI groups (*p* = 0.8964). The above findings implied that GlyNAC enhances the restoration of the grip of the hindlimb in rats after SCI, and that this ameliorative effect is better when BWSTT is used in combination with GlyNAC than when it is used alone.

### 3.4. GlyNAC Improved Motor Neuron Excitability in SCI Model Rats

The effect of GlyNAC on motor neuron excitability and electrophysiology in the control, SCI, and GlyNAC + SCI groups is shown in Figure 4C,D. Two weeks after SCI, the RDD of the control and GlyNAC + SCI groups showed a similar trend of attenuation with increasing stimulation frequency, while the RDD of the SCI group was significantly higher than that of the control group starting from 0.5 Hz (*p* = 0.0023, *p* = 0.0026, *p* = 0.0005, and *p* = 0.0001 for 0.5, 1, 2, and 5 Hz, respectively); however, from 2 Hz, the RDD in the SCI group was significantly higher than that in the GlyNAC+SCI group (*p* = 0.0124 and *p* = 0.0035 for 2 and 5 Hz, respectively); at 4 weeks, the RDD showed a similar trend to that at 2 weeks, namely, it was significantly higher in the SCI group than in the control group from 0.5 Hz onwards (*p* = 0, *p* = 0.0012, *p* = 0.0023, and *p* = 0.0114 for 0.5, 1, 2, and 5 Hz, respectively), but it was significantly higher than that of GlyNAC group at 2 and 5 Hz (*p* = 0.0317 and *p* = 0.0296, respectively); at 6 and 8 weeks, the RDD of the SCI group was significantly higher than that of control group, but it was not significantly different from that of the GlyNAC + SCI group. The above results indicated that the motor neuron excitability and the tone of skeletal muscles had increased in the injured area in SCI model rats, which could lead to spasticity; however, GlyNAC treatment reduced the motor neuron excitability of the skeletal muscles in a short time, thereby reducing the skeletal muscle tone and improving skeletal muscle physiological function.

### 3.5. Effects of GlyNAC Combined with BWSTT on Neurons and Motor Neurons at the Anterior Horn of the Lumbar Enlargement of the Spinal Cord

**Figure 5:**
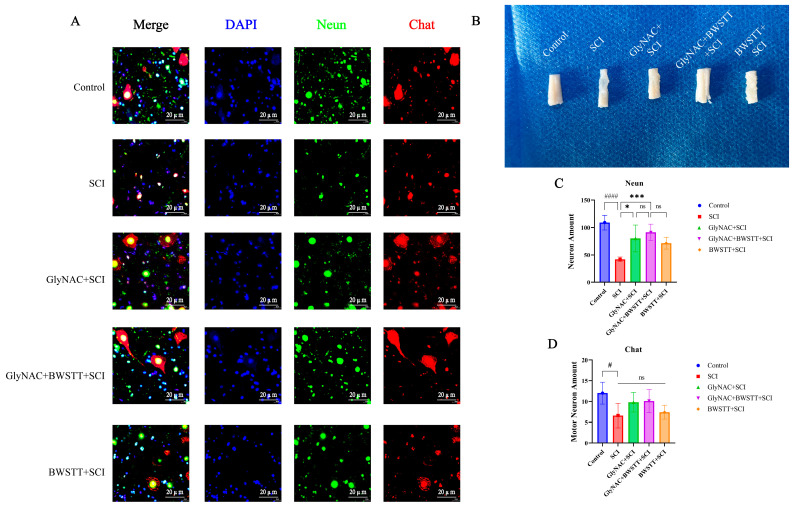
Lumbar enlargement neurons, motor neurons, and morphological observation. (**A**) Immunofluorescence staining for NeuN and ChAT in the lumbar enlargement of the control, SCI, GlyNAC + SCI, GlyNAC + BWSTT + SCI, and BWSTT + SCI groups. (**B**) Morphological observation of the median spinal cord injury site in the different groups. (**C**,**D**) Number of neurons and motor neurons in the different groups. * indicates significant differences between the SCI group and the GlyNAC + SCI, GlyNAC + BWSTT + SCI, and BWSTT + SCI groups, # indicates significant differences between the control group and the SCI group, and ns denotes not significant (one-way ANOVA, Bonferroni post hoc test). * and # indicate *p* < 0.05. *** indicate *p* < 0.001. #### indicate *p* < 0.0001. GlyNAC: glycine and N-acetylcysteine; SCI: spinal cord injury; BWSTT: body weight support treadmill training.

The number of neurons, including motor neurons, in the anterior horn of the lumbar enlargement of the spinal cord was significantly greater in the GlyNAC + SCI and GlyNAC + BWSTT + SCI groups than in the SCI group (Figure 5A). As shown in Figure 5B, morphologically, spinal cord tissues in the median part of the injury in SCI rats exhibited obvious atrophy; in comparison, spinal cord tissue atrophy was significantly reduced in rats of both the GlyNAC + SCI and GlyNAC + BWSTT + SCI groups, suggesting that GlyNAC exerted neuroprotective effects against the injured spinal cord, at least to a certain extent, and alleviated secondary injury. Further analysis (Figure 5C,D) indicated that the number of neurons in the anterior horn of the lumbar enlargement in the SCI group (41.9 ± 4.2778) was significantly decreased compared with that in the control group (109.1 ± 13.3716; *p* < 0.0001), the GlyNAC + SCI group (80 ± 24.551; *p* = 0.0137), and the GlyNAC + BWSTT + SCI group (91.2 ± 14.9399; *p* = 0.0004); however, there was no significant difference in the number of neurons between the GlyNAC + BWSTT + SCI group (91.2 ± 14.9399) and the GlyNAC + SCI group (80 ± 24.551; *p* = 0.5047) or the BWSTT + SCI group (71.4 ± 10.8132; *p* = 0.1933). We further found that the number of motor neurons was significantly decreased in the SCI group compared with that in the control group (*p* = 0.0386), but it did not significantly differ from that of the other groups. In conclusion, the number of neurons and motor neurons was significantly decreased in the lumbar enlargement area of SCI model rats; however, while GlyNAC administration reduced neuronal death, it did not show a significant protective effect on motor neurons, suggesting that GlyNAC has a good neuroprotective effect, but only to a certain extent.

### 3.6. Therapeutic Effects of GlyNAC Combined with BWSTT on Skeletal Muscle Fiber CSA and Density

**Figure 6:**
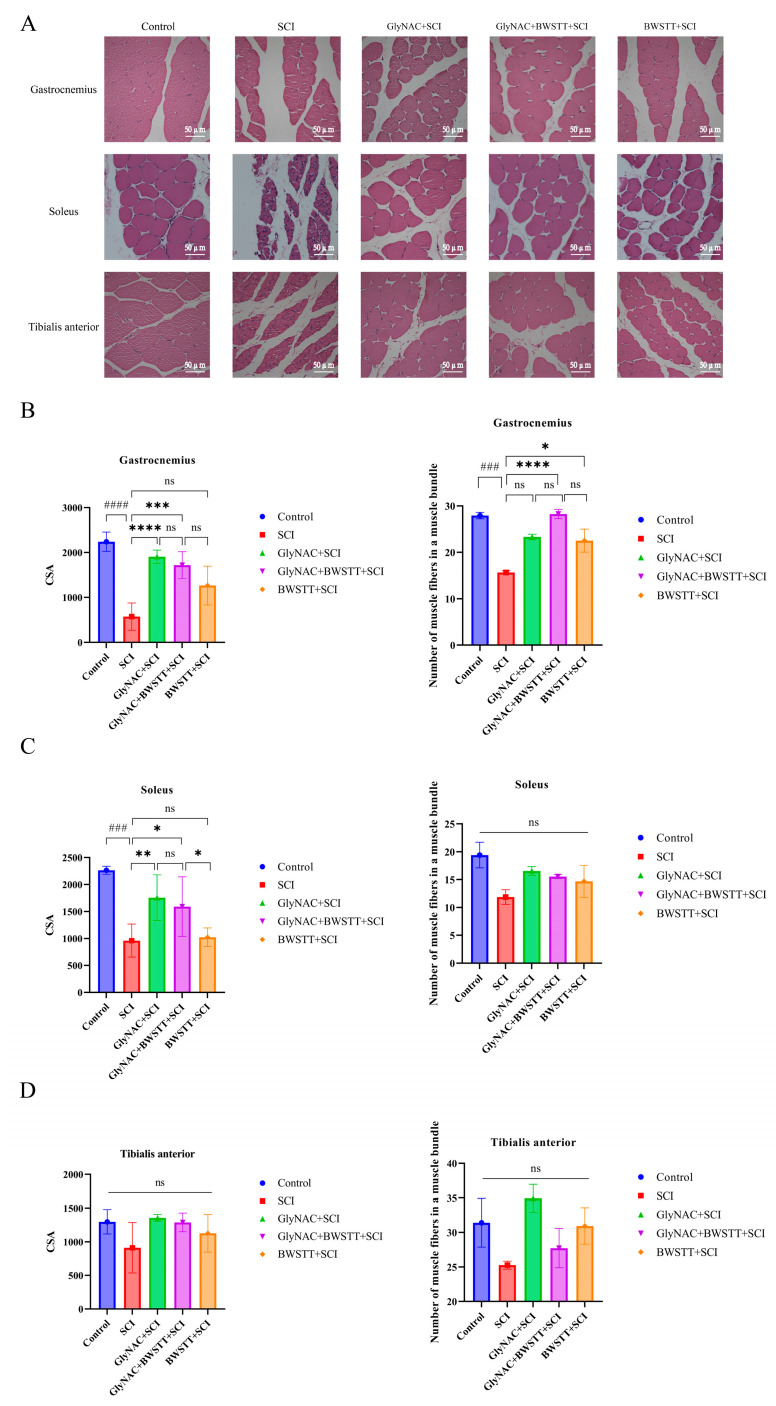
Morphological observation of H&E-stained muscle fibers from the gastrocnemius, soleus, and tibialis anterior muscles and the results of the data analysis relating to muscle fiber CSA and density. (**A**) H&E staining of gastrocnemius, soleus, and tibialis anterior in the control, SCI, GlyNAC + SCI, GlyNAC + BWSTT + SCI, and BWSTT + SCI groups. (**B**) Results of the analysis of CSA and density of muscle fibers in muscle bundles of gastrocnemius fibers in the different groups. (**C**) Results of the analysis of the CSA and density of muscle fibers in muscle bundles of the soleus in the different groups. (**D**) Results of the analysis relating to the CSA and density of muscle fibers in muscle bundles of the tibialis anterior in the different groups. * indicates significant differences between the GlyNAC + SCI, GlyNAC + BWSTT + SCI, and BWSTT + SCI groups and the SCI group, # indicates significant differences between the control group and the SCI group, and ns denotes not significant (one-way ANOVA, Bonferroni post hoc test). * indicates *p* < 0.05. ** indicates *p* < 0.01. *** and ### indicate *p* < 0.001. **** and #### indicate *p* < 0.0001. CSA, cross-sectional area; H&E: hematoxylin and eosin; GlyNAC: glycine and N-acetylcysteine; SCI: spinal cord injury; BWSTT: body weight support treadmill training.

As shown in Figure 6A, the myocytes of the gastrocnemius, soleus, and tibialis anterior muscles in the control group were regular in shape, tightly arranged, with nuclei regularly distributed in the submuscular membrane, and with no necrosis and apoptosis of skeletal muscle cells, while the skeletal muscle cells of the SCI group showed obvious atrophy, degeneration and necrosis, enlarged cellular gaps, and were loosely arranged; the atrophy and necrosis of the myocytes was significantly alleviated when the intervention of GlyNAC and BWSTT was given, with myocytes more tightly arranged than those of the SCI group, and the improvement was even more pronounced with the combined intervention of GlyNAC and BWSTT. Next, we undertook an analysis of the CSA of skeletal muscle fibers and the density of muscle fibers in bundles of muscles below the injury area that have important roles during exercise—the gastrocnemius, soleus, and tibialis anterior. H&E staining results of the three muscles in the five groups of rats demonstrated that the degree of muscle fiber atrophy was significantly lower in the GlyNAC and BWSTT intervention groups than in the SCI group (Figure 6A).

As shown in Figure 6B, the CSA of muscle fibers in the gastrocnemius of SCI rats was significantly decreased compared with that in rats in the control, GlyNAC + SCI, and GlyNAC + BWSTT + SCI groups (*p* < 0.0001, *p* = 0, and *p* = 0.0005, respectively); however, no significant difference in muscle fiber CSA was detected between the GlyNAC + BWSTT + SCI group and the GlyNAC + SCI group or between the GlyNAC + BWSTT + SCI group and the BWSTT + SCI group (*p* = 0.78 and *p* = 0.1121, respectively). Meanwhile, the density of muscle fibers in bundles of the gastrocnemius of SCI rats was significantly decreased compared with that in rats of the control, GlyNAC + BWSTT + SCI, and BWSTT + SCI groups (*p* = 0.0002, *p* = 0.0001, and *p* = 0.0406, respectively); no difference in muscle fiber density was observed between the GlyNAC + BWSTT + SCI group and the GlyNAC + SCI or BWSTT + SCI groups (*p* = 0.0541 and *p* = 0.1153, respectively).

The CSA of muscle fibers in the soleus of SCI model rats was significantly decreased compared with that in animals of the control, GlyNAC + SCI, and GlyNAC + BWSTT + SCI groups (*p* = 0.0001, *p* = 0.0079, and *p* = 0.0281, respectively) (Figure 6C); additionally, the CSA of soleus muscle fibers was significantly decreased in the BWSTT + SCI group compared with that in the GlyNAC + BWSTT + SCI group (Figure 6C); however, there was no significant difference in the CSA of soleus muscle fibers between the GlyNAC + BWSTT + SCI and GlyNAC + SCI groups (*p* = 0.9764). There was no significant difference in soleus muscle fiber density among the five groups (Figure 6C). Similarly, no significant difference was seen in the CSA or density of myofibers in the tibialis anterior among the five groups of rats (Figure 6D).

Overall, these findings showed that GlyNAC was effective in delaying gastrocnemius and soleus atrophy after SCI; however, no additional therapeutic effect relating to the maintenance of myofiber CSA or myofiber density in skeletal muscle was gained when GlyNAC treatment was combined with BWSTT.

### 3.7. GlyNAC Combined with BWSTT Protects Motor Endplates in the Gastrocnemius after SCI

**Figure 7:**
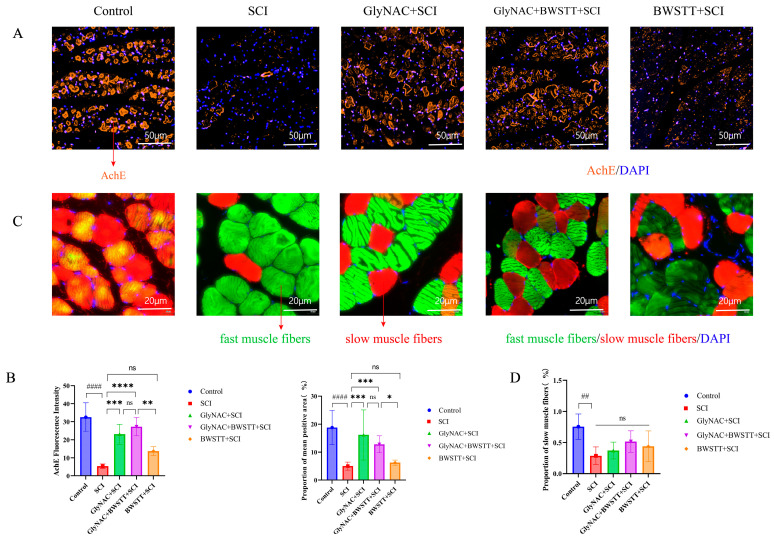
AChE immunofluorescence staining in the gastrocnemius, fast and slow muscle staining in the soleus, and the results of data analysis. (**A**) AChE staining in the gastrocnemius in the control, SCI, GlyNAC + SCI, GlyNAC + BWSTT + SCI, and BWSTT + SCI groups. (**B**) Results of the data analysis for immunofluorescence intensity and mean AChE-positive area in the different groups. (**C**) Staining of fast and slow soleus muscle fibers in the different groups (fast muscle fibers are shown in green, slow muscle fibers are shown in red). (**D**) Results of the data analysis relating to the proportion of slow muscle fibers in the soleus in the different groups. * indicates significant differences between the SCI group and the GlyNAC + SCI, GlyNAC + BWSTT + SCI, and BWSTT + SCI groups; # indicates significant differences between the control group and the SCI group; and ns denotes not significant (one-way ANOVA, Bonferroni post hoc test). * indicates *p* < 0.05. ** and ## indicate *p* < 0.01. *** indicates *p* < 0.001, **** and #### indicate *p* < 0.0001. AChE: acetylcholinesterase; GlyNAC: glycine and N-acetylcysteine; SCI: spinal cord injury; BWSTT: body weight support treadmill training.

In this study, we used acetylcholinesterase (AChE) as a motor endplate marker. As shown in Figure 7A, AChE staining was more complete, clearer, and substantially more extensive in the GlyNAC + SCI and GlyNAC + BWSTT + SCI groups compared with that in the SCI and BWSTT + SCI groups. The fluorescence intensity and the proportion of the mean AChE-positive area among the five groups are shown in Figure 7B. The AChE immunofluorescence intensity in the gastrocnemius of rats in the SCI group was significantly lower than that of the control, GlyNAC + SCI, and GlyNAC + BWSTT + SCI groups (*p* < 0.0001, *p* = 0.0002, and *p* < 0.0001, respectively), and it was also significantly lower in the BWSTT + SCI group than in the GlyNAC + BWSTT + SCI group (*p* = 0.002); however, there was no significant difference in AChE immunofluorescence intensity in the gastrocnemius between the GlyNAC + SCI and GlyNAC + BWSTT + SCI groups (*p* = 0.7173). The proportion of the mean AChE-positive area in the gastrocnemius was significantly lower in the SCI group than in the control, GlyNAC + SCI, and GlyNAC + BWSTT + SCI groups (*p* < 0.0001, *p* = 0.0003, and *p* = 0.0007, respectively), and it was also significantly lower in the BWSTT + SCI group than in the GlyNAC + BWSTT + SCI group (*p* = 0.0127); however, no significant difference in this parameter was observed between the GlyNAC + SCI and GlyNAC + BWSTT + SCI groups (*p* = 0.9974). The above results indicated that GlyNAC can help protect the motor endplate in the gastrocnemius muscle of rats after SCI, thus contributing to the maintenance of nerve conduction in this muscle.

### 3.8. The Effect of GlyNAC in Combination with BWSTT on the Conversion of Fast and Slow Muscle Fibers in the Soleus

By visualization of immunofluorescence-stained sections, there was a significant increase in fast muscle fibers and a significant decrease in slow muscle fibers in the SCI group compared with that in the control group, whereas there was a significant increase in slow muscle fibers in the GlyNAC + BWSTT + SCI group relative to that in the SCI group (Figure 7C). As can be seen in Figure 7D, the proportion of slow muscle fibers in the soleus was significantly lower (*p* = 0.0097) in rats of the SCI group (0.2855 ± 0.1426) than in those of the control group (0.755 ± 0.2048); however, the proportion of slow muscle fibers in the GlyNAC + SCI (0.371 ± 0.1353), GlyNAC + BWSTT + SCI (0.5151± 0.1738), and BWSTT + SCI groups (0.4383 ± 0.1738) did not differ significantly from that seen in the SCI group (*p* = 0.9295, *p* = 0.2316, and *p* = 0.5856, respectively).

The above observations show that there is a significant decrease in the proportion of slow muscle fibers in the soleus of model rats after SCI; although the differences in the proportions of muscle fibers between the intervention groups did not reach significance, the results nevertheless suggested that the combination treatment may have a better therapeutic effect than single treatment in inhibiting slow-to-fast muscle fiber conversion.

## 4. Discussion

GSH plays a crucial role in antioxidant defenses and the concentration of GSH is affected after SCI. A reduction in GSH concentrations in the spinal cord leads to a decrease in antioxidant capacity, resulting in further oxidative stress-induced damage [23]. GSH is synthesized from glutamate, glycine, and cysteine [24]. SCI leads to the activation of glutamate receptors and greatly increased glutamate concentrations [25], followed by a decrease in the ability of mitochondria to maintain homeostasis. This results in ATP-dependent cellular excitotoxicity as well as excessive reactive oxygen species (ROS) accumulation, with a consequent increase in oxidative stress-induced damage [26]. Serum and spinal cord levels of glycine are decreased after SCI. Glycine can be supplemented orally [27] and exerts an inhibitory effect against skeletal muscle atrophy resulting from oxidative stress-related injury and inflammation [28]. Glycine supplementation is effective at restoring both normal anabolic responses in muscle and the role of leucine in delaying muscle proteolysis [29]. Sun et al. [30] found that glycine regulates protein turnover in skeletal muscle by activating Akt/mTOR and inhibiting the expression of the atrophy-associated genes MuRF-1 and Atrogin-1 in myofibroblasts. N-Acetylcysteine (NAC) is a source of sulfhydryl groups and has more potent antioxidant activity than cysteine. It is one of the most potent ROS scavengers and reverses oxidative stress damage that would otherwise lead to mitochondrial dysfunction and neurological deficits [31,32,33,34]. Paz García-Campos [35] found that NAC improves oxidative homeostasis in skeletal muscle and increases skeletal muscle strength, thereby counteracting dystrophies; this suggests that NAC has good therapeutic potential against skeletal muscle atrophy.

Kumar et al. [18,19,36] found that GlyNAC was effective at increasing blood GSH levels in elderly subjects, thereby protecting against aging-induced oxidative damage; increasing grip strength levels in elderly subjects; decreasing the rate of protein breakdown in skeletal muscle; and counteracting oxidative stress-related damage in patients with COVID-19. Sekhar [37] showed that GlyNAC improved mitochondrial dysfunction and attenuated insulin resistance in type II diabetes. Given the documented ability of GlyNAC to protect against oxidative stress-induced damage, we hypothesized that GlyNAC therapy may have the potential to maintain skeletal muscle mass and delay skeletal muscle atrophy after SCI. Meanwhile, BWSTT is an exercise-based treatment widely used for enhancing the exercise capacity of patients with SCI. BWSTT can alleviate muscle atrophy after SCI to some extent, improve skeletal muscle sensitivity, and reduce post-injury insulin resistance [38,39,40]. However, in many patients, the effects of braking and poor exercise are not conducive to delaying atrophy in skeletal muscles.

We found that, following GlyNAC administration, rats had significantly higher BBB scores but did not show better motor function when GlyNAC was combined with BWSTT. When we compared gait data among the control, SCI, and GlyNAC groups, we found that gait symmetry was significantly better in the GlyNAC group than in the SCI group, with the results indicating that GlyNAC promoted the recovery of motor function in the rats; we further compared whether combined intervention with GlyNAC and BWSTT exerted a better therapeutic effect than GlyNAC alone, and the results showed that at a speed of 10 cm/s, there was a significant difference in hind stance width between the two groups at 4 weeks post-injury, which we hypothesized might be due to an increase in muscle tone in the rats of the BWSTT group. The results of the 4-week open field test showed that the overall velocities of rats in the GlyNAC + SCI, GlyNAC + BWSTT + SCI, and BWSTT + SCI groups were significantly greater than those of rats in the SCI group, while rats in the GlyNAC + BWSTT + SCI and BWSTT + SCI groups walked significantly longer distances overall than rats in the SCI group, indicating that BWSTT significantly improved the athletic endurance of rats after SCI; rats in the GlyNAC + SCI and BWSTT + SCI groups crossed the central area of the open field substantially more frequently than those in the SCI group, indicating that the two interventions improved the emotional state of the rats to different degrees and increased their motivation to exercise; however, at 8 weeks, the overall velocities and distances traveled among rats of the GlyNAC + BWSTT + SCI group were significantly greater than those of the SCI group and the two single intervention groups. This indicated that the GlyNAC/BWSTT combination was more effective at promoting the recovery of voluntary motor function and improving the athletic endurance of the rats after SCI, effects that lasted long after the treatment had stopped. Hindlimb grip can objectively reflect the strength of the hindlimbs and the grip strength of the rats when walking. At 4 weeks post-injury, the grip strength of the hindlimbs of rats in the GlyNAC + BWSTT + SCI group was significantly greater than that of rats in the SCI group and the two single intervention groups; at 8 weeks, the grip strength of the hindlimbs of rats in the GlyNAC + BWSTT + SCI group was significantly greater than that of rats of the SCI and BWSTT + SCI groups, which indicated that the combination treatment significantly enhanced the hindlimb grip strength of rats after SCI, and the therapeutic effect was better than that of the two respective individual interventions. The results of the above studies demonstrated that GlyNAC can significantly improve voluntary motor function in rats, while BWSTT can enhance exercise endurance; when combined, GlyNAC and BWSTT can significantly improve the voluntary motor ability of rats following SCI and for extended periods.

SCI predisposes patients to upper motor neuron damage which triggers spinal motor neuron excitation and leads to spasticity and elevated muscle tone which are prevalent in patients with cervical, thoracic, and lumbar medullary injuries [41,42,43,44], which severely affects their functional recovery and reduces their quality of life [45]. An increase in tension is common in patients with cervical and lumbar cord injuries [38], while elevated skeletal muscle tone after SCI has been shown to have beneficial effects by preserving skeletal muscle properties and attenuating the slow-to-fast conversion of myosin [46,47]. We examined neuron excitability in rats of the control, SCI, and GlyNAC + SCI groups using electrophysiological assays and found that the RDD of the GlyNAC + SCI group showed a decreasing trend with the gradual increase in the stimulation frequency compared with that in SCI group at 2 and 4 weeks post-surgery, an effect that gradually disappeared after the discontinuation of GlyNAC administration (at the 6- and 8-week sampling points). This suggested that GlyNAC has a short-term effect in inhibiting motor neuron excitability and improving skeletal muscle spasticity.

The damaged spinal cord area after SCI is subjected to multiple injuries from both primary damage and secondary damage due to inflammation and oxidative stress, among other factors. This can result in irreversible neuronal damage, finally leading to apoptosis [48,49] with a concomitant reduction in the number of neurons at the site of injury in the spinal cord, including cholinergic neurons, which are closely related to motor function. Cholinergic neurons play an important role in skeletal muscle innervation. They innervate the neuromuscular junction (NMJ), leading to the release of the neurotransmitter acetylcholine (ACh) and, consequently, depolarization of muscle fibers. However, SCI can damage cholinergic neurons, which impairs nerve conduction in skeletal muscle [5,50,51]. In this study, the anterior horn of the lumbar enlargement, which is closely related to the motor function of the lower limbs, was selected as the detection site, with NeuN and choline acetyltransferase (ChAT) serving as markers for neurons and motor neurons, respectively. The results showed that the number of neurons and motor neurons in the lumbar enlargement of the SCI group was significantly decreased compared with that in the control group. Additionally, the number of neurons in the GlyNAC + SCI and GlyNAC + BWSTT + SCI groups was significantly higher than that in the SCI group. Meanwhile, the number of motor neurons was higher in both these groups than in the SCI group, although the difference was not significant. This suggested that GlyNAC exerts a partial neuroprotective effect, thus increasing the survival rate of spinal cord neurons and motor neurons and helping to preserve neural function.

The chronic phase of SCI is primarily characterized by atrophy of the skeletal muscles of distal limbs below the diseased tissue due to a prolonged reduction in distal joint contractility [5]. In our study, three muscles of the lower extremities—the gastrocnemius, soleus, and tibialis anterior—that play key roles in controlling hip, knee, and ankle joint motion, as well as in the walking process, were selected as targets for analysis. Through the observation of the HE staining of skeletal muscle, it can be found that GlyNAC and BWSTT can effectively counteract myocyte atrophy and necrosis and restore the alignment of myocytes, and the combination of the two interventions is more effective, which was also verified by quantitative analysis. Results at 8 weeks post-injury showed that the CSA of the gastrocnemius and soleus in the SCI group was significantly reduced compared with that in the control group, and the CSA of the gastrocnemius and soleus was significantly greater in the GlyNAC + SCI and GlyNAC + BWSTT + SCI groups than in the SCI group. Furthermore, the density of the muscle fibers in the gastrocnemius bundles was significantly lower in the SCI group than in the control, GlyNAC + BWSTT + SCI, and BWSTT + SCI groups. There was no significant difference in CSA or muscle fiber density in the tibialis anterior among the five groups. Muscle fiber analysis showed that GlyNAC can effectively delay skeletal muscle atrophy and that the GlyNAC/BWSTT combination treatment was more effective at maintaining the structure and morphology of skeletal muscle fibers than either treatment alone.

The NMJ, also known as the motor endplate, is the point of contact of axon endings of motor neurons in the anterior horn of the spinal cord on skeletal muscle fibers and is a specialized chemical synapse that regulates physiological functions and nourishes skeletal muscle. The presynaptic component is the axon ending and the postsynaptic component is the receptor for the neurotransmitter acetylcholine. AChE, located in the cholinergic synaptic gap, is a key enzyme in biological nerve conduction. It catalyzes the degradation of acetylcholine, thereby terminating the excitatory effect of the neurotransmitter on the postsynaptic membrane and ensuring the normal transmission of neural signals. AChE is a recognized marker of motor endplate degeneration, recovery, and regeneration [52,53,54]. Here, we performed immunofluorescence staining for AChE in the gastrocnemius at 8 weeks post-injury. We found that the intensity of AChE immunofluorescence and the percentage of the AChE-positive area were significantly greater in the GlyNAC + SCI and GlyNAC + BWSTT + SCI groups than in the SCI group or the BWSTT + SCI group, which suggested that GlyNAC protects the motor endplates of the skeletal muscle from degeneration after SCI, thus helping to preserve skeletal muscle nerve conduction and nutritional function.

SCI promotes a shift in skeletal muscle fiber type, namely, a conversion from slow to fast muscle fibers, resulting in a decrease in skeletal muscle endurance and strength. The soleus is a key muscle of the lower extremity that is dominated by slow muscle fibers, and a decrease in the proportion of slow muscle fibers is often observed in the soleus muscle after SCI [55,56]. Our data showed that the proportion of slow muscle fibers in the SCI group was significantly lower than that in the control group. Additionally, although the proportion of slow muscle fibers did not differ significantly between the SCI group and the three other groups, the value of this parameter tended to be higher in the latter than in the former. We speculate that this lack of a significant inhibitory effect on muscle fiber type conversion can be explained by the short duration of the experimental period and the small sample size. However, further studies are needed to confirm this possibility.

Both somatic and visceral nerve function are affected after SCI [1]. In addition, SCI leads to a decrease in limb motor function, resulting in limb braking and wasting muscle atrophy, as well as the secretion of hormones that regulate and maintain skeletal muscle mass [57,58,59]. Skeletal muscle atrophy, a common complication of SCI, is exacerbated by a combination of factors. For example, it has been found that protein expression and mitochondrial function in skeletal muscle are impaired after SCI [60]. The subsequent increase in the release of ROS leads to the ubiquitination and degradation of proteins in skeletal muscle and results in damage to the structure of the motor endplate, motor endplate dysfunction, accumulation of fat in the skeletal muscle, type II diabetes, and cardiovascular disease, among other conditions [61,62]. This highlights the importance of determining the underlying causes of skeletal muscle atrophy after SCI and identifying strategies for its prevention.

Skeletal muscle after SCI is currently treated with exercise training, physical therapy, and testosterone therapy, among other therapeutic options, but each has its advantages and disadvantages in delaying skeletal muscle atrophy [61,63]. Although many studies, both basic and clinical, have been conducted relating to the treatment of post-SCI skeletal muscle atrophy, no drug that can counteract skeletal muscle atrophy after severe SCI has yet been approved [4]. The hormone testosterone has a well-documented role in the maintenance of muscle healing. At low doses, testosterone has not been found to be effective in combating post-SCI skeletal muscle atrophy in related studies [64,65]. Although it can be effective in delaying post-SCI skeletal muscle atrophy at moderate doses [66], testosterone treatment is associated with side effects, such as prostate hypertrophy [11]. Wu et al. [67] found that nandrolone, in combination with physiological doses of testosterone, could maintain skeletal muscle mass and promote the normalization of muscle fiber types after SCI. Consequently, the specific role and dosage of testosterone need to be further clarified, and it may need to be combined with other methods of treatment to mitigate its side effects. Myatich et al. [68] highlighted the potential of clemastine as a treatment for SCI-induced skeletal muscle atrophy through the promotion of myelination. Meanwhile, Scholpa et al. [60] reported that the β2-adrenergic receptor agonist formoterol could delay skeletal muscle atrophy by inducing mitochondrial biogenesis. Many drugs targeted at delaying or treating skeletal muscle atrophy are at the basic research stage, and their safety and efficacy in the clinic need to be verified. Within this context, in the present study, we assessed whether the exogenous administration of two amino acids, glycine and N-acetylcysteine, combined with a more generalized clinical BWSTT, could improve or delay skeletal muscle atrophy after SCI.

### Research Innovations and Limitations

GlyNAC has not been investigated in the treatment of skeletal muscle atrophy in SCI. Here, we assessed its putative protective effect on the structure and function of the spinal cord, spinal cord skeletal muscle, and the motor endplate of skeletal muscle. Additionally, we combined GlyNAC administration with BWSTT, which has widespread clinical use, aiming to provide an informative “exercise + nutrition” clinical treatment model for skeletal muscle atrophy in patients with SCI, and further clinical studies are needed in the future. However, the study duration was relatively short, and it is thus not possible to provide evidence for the long-term effects of the interventions used in this study. Furthermore, the training method used here was relatively homogeneous, with only weight-loss walking training being employed. Multi-modal exercise training will be required if a more efficient and comprehensive treatment program is to be developed.

## 5. Conclusions

GlyNAC can effectively protect the structure and function of the spinal cord and skeletal muscle, improve motor function, reduce muscle tone and spasticity, and improve the strength of the hindlimbs of rats following SCI; meanwhile, the combination of GlyNAC and BWSTT showed a better therapeutic effect on voluntary motor function and also improved the structure and function of the skeletal muscle of rats with SCI.

## Figures and Tables

**Table 1:** The primary antibodies used in this study.

Antibody	Host	Supplier	Catalog No.	Dilution	Application
ChAT	Rabbit	Abcam, Cambridge, UK	ab181023	1:1000	IF
NeuN	Rabbit	Servicebio	GB11138	1:200	IF
Fast	Rabbit	Servicebio	GB112130	1:3000	IF
Slow	Rabbit	Servicebio	GB112131	1:500	1:500
AChE	Rabbit	Servicebio	GB11038-1	1:500	1:500

## Data Availability

The datasets and materials used and/or analyzed during the current study are available from the corresponding author on reasonable request.
